# Mechanism of Protection by Soluble Epoxide Hydrolase Inhibition in Type 2 Diabetic Stroke

**DOI:** 10.1371/journal.pone.0097529

**Published:** 2014-05-13

**Authors:** Kristen L. Zuloaga, Stephanie M. Krasnow, Xinxia Zhu, Wenri Zhang, Sari A. Jouihan, Robert E. Shangraw, Nabil J. Alkayed, Daniel L. Marks

**Affiliations:** 1 The Knight Cardiovascular Institute, Department of Anesthesiology & Perioperative Medicine, Oregon Health & Science University, Portland, Oregon, United States of America; 2 Papé Family Pediatric Research Institute, Department of Pediatrics, Oregon Health and Science University, Portland, Oregon, United States of America; University of Münster, Germany

## Abstract

Inhibition of soluble epoxide hydrolase (sEH) is a potential target of therapy for ischemic injury. sEH metabolizes neuroprotective epoxyeicosatrienoic acids (EETs). We recently demonstrated that sEH inhibition reduces infarct size after middle cerebral artery occlusion (MCAO) in type 1 diabetic mice. We hypothesized that inhibition of sEH would protect against ischemic injury in type 2 diabetic mice. Type 2 diabetes was produced by combined high-fat diet, nicotinamide and streptozotocin in male mice. Diabetic and control mice were treated with vehicle or the sEH inhibitor t-AUCB then subjected to 60-min MCAO. Compared to chow-fed mice, high fat diet-fed mice exhibited an upregulation of sEH mRNA and protein in brain, but no differences in brain EETs levels were observed between groups. Type 2 diabetic mice had increased blood glucose levels at baseline and throughout ischemia, decreased laser-Doppler perfusion of the MCA territory after reperfusion, and sustained larger cortical infarcts compared to control mice. t-AUCB decreased fasting glucose levels at baseline and throughout ischemia, improved cortical perfusion after MCAO and significantly reduced infarct size in diabetic mice. We conclude that sEH inhibition, as a preventative treatment, improves glycemic status, post-ischemic reperfusion in the ischemic territory, and stroke outcome in type 2 diabetic mice.

## Introduction

Individuals with diabetes have more than twice the risk for stroke compared to non-diabetic individuals [1]. Hyperglycemia is also associated with poor stroke outcome in both humans [2]–[4] and in several rodent models of stroke [5]–[10]. Approximately 40% of ischemic stroke patients are hyperglycemic upon admission to the hospital [4]. Clinically, blood glucose levels correlate with both infarct size and degree of disability [4]. However, tight glycemic control in hyperglycemic patients has failed to protect against stroke incidence or improve outcome in clinical trials [11]–[16]. Since tight glycemic control has failed to protect hyperglycemic patients from increased stroke risk and worse stroke outcome, the goal of the current study was to determine if inhibition of soluble epoxide hydrolase (sEH) would protect against ischemic injury in type 2 diabetic mice. sEH is a potential mediator of ischemic injury via its metabolism of neuroprotective epoxyeicosatrienoic acids (EETs). sEH is expressed in a variety of cells in the brain including cerebrovascular endothelium, vascular smooth muscle cells, neurons, oligodendrocytes, and astrocytes [17].

Using a rodent model of type 1 diabetes, we have recently shown that hyperglycemia decreases brain EETs concentrations and increases infarct size after MCAO [8]. Furthermore, we showed that sEH inhibition could restore brain EETs concentrations and reduce infarct size in type 1 diabetic mice [8]. While both type 1 and 2 diabetes mellitus are characterized by hyperglycemia, the two diseases are metabolically quite distinct. Type 1 diabetes results in hyperglycemia due to destruction of pancreatic beta cells leading to absolute insulin deficiency. In contrast type 2 diabetes results in hyperglycemia due to insulin resistance or relative insulin deficiency, and is commonly associated with obesity, dyslipidemia, and hypertension [18]. In the current study, we wanted to determine whether the protective effect of sEH inhibition would extend to the setting of type 2 diabetes, a much more prevalent and complex hyperglycemic disease. In addition, we utilized a rodent model of pre-diabetes to determine if sEH is upregulated before development of overt type 2 diabetes. We hypothesized that inhibition of sEH, as a preventative treatment, would protect against ischemic injury in type 2 diabetic mice.

## Materials and Methods

### Ethics Statement

Our study was conducted in accordance with National Institutes of Health guidelines for care and use of animals in research and conformed to the Association for Assessment and Accreditation of Laboratory Animal Care AAALAC Accreditation and the Office of Laboratory Animal Welfare (OLAW Assurance #A3304-01, approved June 2012). All protocols were approved by the Institutional Animal Care and Use Committee of Oregon Health & Science University (Portland, OR).

### High Fat Diet Model of Pre-diabetes in Mice

Long-term high fat diet is a model of pre-diabetes in mice, leading to elevated body weight and impaired glucose tolerance without causing overt hyperglycemia [19]. Five-week old male C57BL/6J mice (JAX) were acclimatized to the animal facility and then placed on a high fat (60% fat) diet (D12492, Research Diets, Inc., New Brunswick, NJ) or normal chow (13% fat) diet (LabDiet 5001; Nestle Purina, St. Louis, MO) for 15 weeks. Weight was tracked biweekly. At 20 weeks of age, mice were fasted overnight then subjected to a glucose tolerance test (GTT). For the GTT, blood glucose was measured just prior to injection of glucose (2 g/kg, i.p.), and once every 15–30 minutes for 2 hrs after the injection. Insulin levels were measured by radioimmunoassay using a Rat Insulin RIA Kit (Millipore, Billerica, MA). Measurements were run in duplicate and performed according to the manufacturer’s instructions. The intra-assay coefficient of variation was 5.7%.

### High Fat Diet, Streptozotocin and Nicotinamide (HFD+STZ/NA) Model of Type 2 Diabetes in Mice

Five-week old male C57BL/6J mice (JAX) were acclimatized to the animal facility and placed on a high fat (60% fat) diet (D12492, Research Diets, Inc., New Brunswick, NJ) or normal chow (13% fat) diet (LabDiet 5001; Nestle Purina, St. Louis, MO) for 4 wks. After 4 wks on the high fat diet, mice were fasted overnight and treated with nicotinamide (NA; 240 mg/kg, i.p.) and streptozotocin (STZ; 100 mg/kg, i.p.) 15 min later. Chow-fed controls received equal volume of saline (i.p.) followed by 50 mM citric acid buffer (i.p) 15 min later. Two days later, the mice were again fasted and treated with either NA/STZ or saline/citric acid buffer. Nakamura et al. [1] demonstrated that with STZ treatment alone (type 1 diabetes model) pancreatic beta cells are lost, but that STZ-induced beta cell damage is attenuated by NA in a dose-dependent manner [20]. After 6 weeks on their diets, mice were fasted and glycemia in saphenous vein blood was measured by glucometer (Breeze 2, Bayer, Tarrytown, NY) to confirm hyperglycemia in type 2 diabetic mice. This model of type 2 diabetes has been well characterized and has been shown to cause insulin resistance [20].

### Inhibition of Soluble Epoxide Hydrolase (sEH) Activity

In vivo sEH activity was inhibited with trans-4-[4-(3-Adamantan-1-yl-ureido) -cyclohexyloxy]-benzoic acid (t-AUCB), developed and generously provided by Dr. Bruce Hammock, University of California, Davis, CA [21]. Mice received 1 mg/kg or 2 mg/kg t-AUCB or saline vehicle i.p. daily for the final 6 days before cerebral ischemia, with the final t-AUCB dose administered immediately after reperfusion.

### Middle Cerebral Artery Occlusion (MCAO)

MCAO was performed 2 weeks after completion of STZ/NA treatment or vehicle for corresponding controls. Transient (60-min) focal cerebral ischemia was induced in overnight-fasted mice using an intraluminal MCAO technique described previously [22]. Briefly, isoflurane-anesthetized mice were instrumented with a laser-Doppler probe and rectal temperature probe and MCAO ischemia induced via insertion of a silicone-coated 6-0 nylon monofilament into the right internal carotid artery until MCA flow was <20% of baseline. The occluding filament was subsequently withdrawn for reperfusion. Mice were allowed to recover and observed for 1 day, after which they were anesthetized with isoflurane, blood collected, and either sacrificed by decapitation and brains removed for infarct size measurement, or perfused with heparinized ice-cold saline for brain collection for qPCR, and Western blot. Insulin levels were measured in serum by radioimmunoassay using a Rat Insulin RIA Kit (Millipore, Billerica, MA). Measurements were run in duplicate and performed according to the manufacturer’s instructions. The intra-assay coefficient of variation was 5.7%.

### Brain Infarct Size

Infarct size in cerebral cortex, caudate putamen and total hemisphere was measured at 24 h after MCAO in 2-mm thick coronal brain sections using 2,3,5-triphenyltetrazolium chloride (TTC) staining and digital image analysis (SigmaScan Pro 5.0, Aspire Software, Ashburn, VA) as previously described [22]. To account for edema, infarcted area was estimated by subtracting the uninfarcted region in ipsilateral hemisphere from the contralateral hemisphere, and expressing infarct volume as a percentage of the contralateral hemisphere.

### Real-time Quantitative PCR

Levels of EPHX2 (sEH gene) mRNA were determined as previously described [8]. In brief, RNA was isolated using a commercial kit (RNAqueous-Micro, Ambion, Austin, TX), contaminant genomic DNA removed by DNase treatment, and RNA reverse transcribed using a commercial high capacity cDNA archive kit (Applied Biosystems, Carlsbad, CA). Resulting cDNA was amplified using TaqMan Universal PCR amplification in a commercial sequence detection system (ABI Prism 7000, Applied Biosystems, Carlsbad, CA). Quantitative PCR was performed in a 96-well plate using 50 µl total volume, in triplicate. PCR was concurrently run on controls without template to assess DNA contamination and primer-dimer formation. Remaining RNA not reverse transcribed was included to control for genomic DNA amplification. 18S was measured as an internal control using an 18S mRNA control kit (FAM-TAMRA, Eurogentec, Seraing, BEL). EPHX2 primers were purchased as a TaqMan Gene Expression Assay (Invitrogen, Catalog #4351372). All final EPHX2 mRNA levels were normalized to 18S.

### Western Blot

sEH protein expression was measured as previously described [8]. In brief, mice were perfused with ice-cold heparinized saline to remove blood, and brains were collected. Brains were homogenized in lysis buffer, centrifuged, and supernatant collected. Protein samples (40 µg) were separated by gel electrophoresis and then transferred to Polyvinylidene Difluoride (PVDF) membranes. Blots were blocked in 5% dry milk, and incubated at 4°C overnight with a primary rabbit polyclonal antibody against murine sEH (1∶500; Cayman Chemical, Ann Arbor, MI). The signal was visualized using a horseradish peroxidase-linked (HRP) rabbit secondary antibody (1∶1,000; GE Healthcare, Piscataway, NJ) followed by detection using Supersignal chemiluminescent reagents (Thermo Fisher Scientific, Waltham, MA) with a FluorChem FC2 (Protein Simple, Santa Clara, CA). Blots were stripped using Restore Western Blot Stripping Buffer (Thermo Fisher Scientific) and re-probed for beta actin (1∶2,000; Sigma-Aldrich) and followed by HRP mouse secondary antibody (1∶1,000; GE Healthcare) and re-imaged. Densitometry was quantified with AlphaView software (Protein Simple) and sEH protein was normalized relative to beta actin as expressed as a ratio compared to chow-fed mice.

### Statistical Analysis

Data are expressed as mean ± SEM. Groups were compared by t-test for two groups or two-way ANOVA with post hoc Newman-Keuls test for multiple measures with multiple groups using GraphPad Prism (GraphPad Software, Inc, La Jolla, CA). Differences were considered significant at p<0.05.

## Results

### High Fat Diet Increases sEH mRNA and Protein Expression in Brain

**Figure 1:**
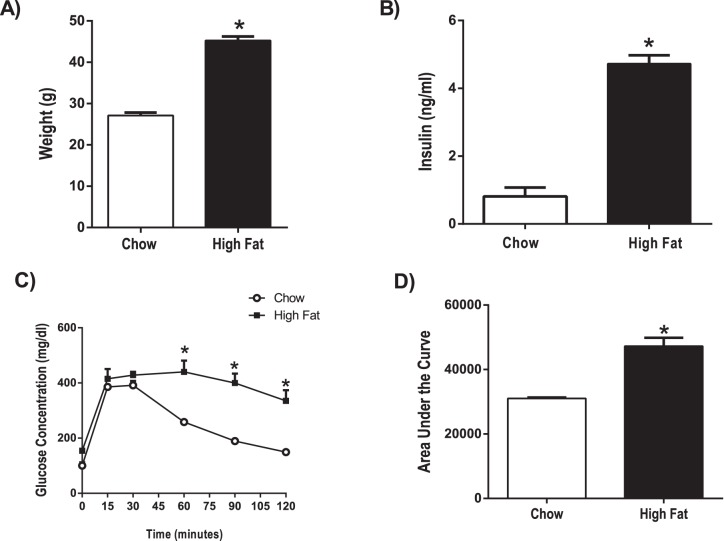
Pre-diabetic mice have increased body weight, increased insulin levels and decreased glucose tolerance. A) Body weight B) serum insulin levels, and C) blood glucose during i.p. glucose tolerance test (GTT) were measured in mice fed a low fat (Chow; n = 5) or high fat (n = 4) diet for 15 weeks. Mice were fasted overnight prior to the GTT. Glucose (2 g/kg body weight) was injected i.p. at time 0. D) Area under the curve from GTT test results was calculated for each treatment group. *p<0.001 vs. Chow.

**Figure 2:**
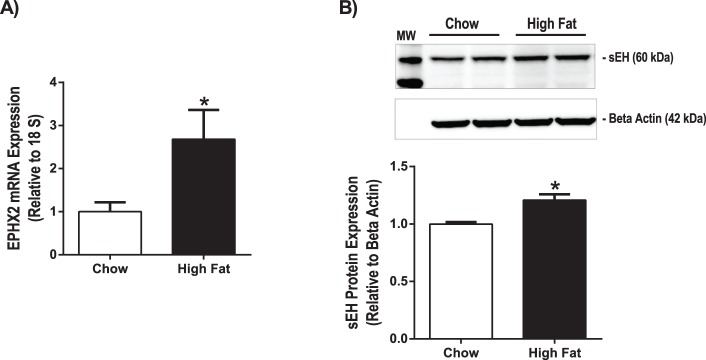
High fat diet increases sEH expression in brain. Mice were treated with a high fat (60% fat) or chow diet (13% fat) for 15 weeks. A) EPHX2 mRNA expression was measured via qPCR in cortex. EPHX2 expression was normalized to 18S expression. *p<0.05 vs. Chow, n = 7 per group. B) sEH protein expression was measured via Western blot in brain. sEH protein expression was normalized to beta actin protein expression. *p<0.01 vs. Chow, n = 4 per group.

Using a rodent model of pre-diabetes, we aimed to determine if sEH expression is altered in the pre-diabetic state, before development of overt type 2 diabetes. The HFD model of pre-diabetes causes impaired glucose tolerance and insulin resistance, without producing the overt hyperglycemia pathognomonic of type 2 diabetes mellitus [19]. Long-term treatment (15 weeks) with a high fat (60% fat) diet caused an increase in body weight (**Fig. 1A**), an increase in insulin levels (**Fig. 1B**) and a decrease in glucose tolerance (**Fig. 1C–D**) in high fat-fed compared to chow-fed mice (p<0.001). **Fig. 2A** shows that, compared to chow-fed mice, high fat diet-fed mice exhibited a 1.7 fold upregulation of EPHX2 (gene encoding for sEH) mRNA in cortex (p<0.05). **Fig. 2B** shows that sEH protein expression was also 20% higher in brains of high fat-diet fed mice compared to chow-fed mice (p<0.01). Despite changes in sEH expression, we did not detect differences in EETs levels, measured via liquid chromatography-tandem mass spectrometry [23], in brains of high fat diet-fed mice compared to chow-fed mice (515+/−62 pg/10 mg wet weight in high fat diet group vs. 399+/−13 pg/10 mg wet weight in chow group, n = 4–5 per group).

### sEH Inhibition Attenuates Increases in Baseline Fasting Blood Glucose Levels in Type 2 Diabetic Mice

**Figure 3:**
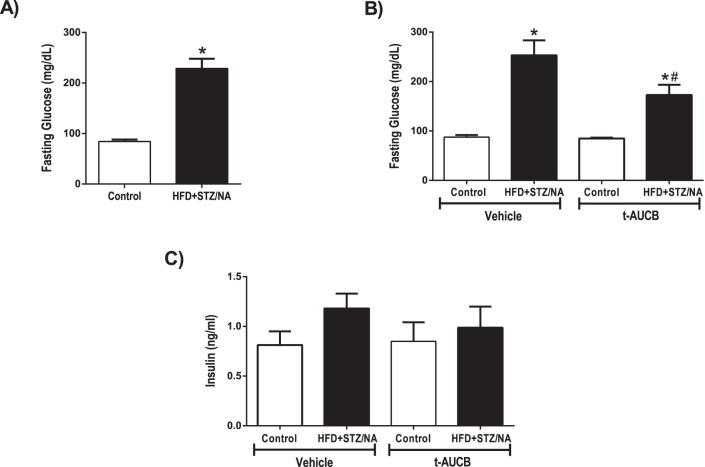
sEH inhibition decreased fasting glucose levels in type 2 diabetic mice. Fasting blood glucose levels were obtained from control and HFD+STZ/NA mice before (A) and after (B) treatment with the sEH inhibitor t-AUCB (1 mg/kg i.p. daily, 7 days) or vehicle. C) Serum insulin levels were also measured in t-AUCB or vehicle treated mice 24 h after ischemia. *p<0.001 vs. Control Vehicle, #p<0.05 vs. HFD+STZ/NA Vehicle. A) n = 20–22 per group. B) n = 5–8 per group. C) n = 2–6 per group.

**Figure 4:**
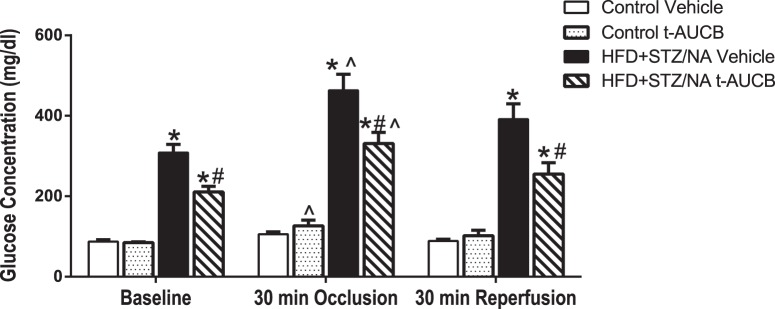
sEH inhibition decreased glucose levels in type 2 diabetic mice before, during, and after ischemia. Control and HFD+STZ/NA mice were treated with the sEH inhibitor t-AUCB (1 mg/kg i.p. daily, 7 days) or vehicle and subjected to middle cerebral artery occlusion (60 min). Blood glucose levels were measured at baseline, 30 min into the occlusion, and 30 min post-reperfusion. *p<0.001 vs. Control Vehicle, #p<0.01 vs. HFD+STZ/NA Vehicle. ∧p<0.05 vs. baseline of same treatment group. N = 4–5 per group.

HFD+STZ/NA was used to model type 2 diabetes. The main endpoint diagnostic criterion for diabetes (either type 1 or type 2) is hyperglycemia [24]. STZ alone induces type 1 diabetes by destroying the pancreatic beta cells, causing insulinopenia and consequent hyperglycemia, and this treatment may also cause a decrease in body weight [20]. Co-administration of NA with STZ attenuates the beta cell injury and prevents overt hyperglycemia [20]. However, if STZ/NA are administered to mice on a high fat diet, the NA is less able to protect the beta cells, resulting in the constellation of hyperglycemia, insulin resistance, and impaired glucose tolerance [20]. This triad is the hallmark of type 2 diabetes [24]. Notably, these treated mice also exhibit hyperlipidemia [20], which is common in type 2 diabetic individuals. **Table 1** shows characteristics of study mice prior to MCAO, which did not differ among groups in age, body weight, or temperature. HFD+STZ/NA mice had increased fasting blood glucose levels compared to control mice at baseline (p<0.001, **Fig. 3A**). The sEH inhibitor t-AUCB reduced fasting blood glucose levels in HFD+STZ/NA mice (p<0.05) but not in control mice (**Fig. 3B**). In addition to baseline blood glucose levels, we also monitored blood glucose levels throughout the MCAO surgery and measured insulin levels 24 h after ischemia. There were no differences in serum insulin levels between groups (**Fig. 3C**). HFD+STZ/NA mice had increased blood glucose levels compared to control mice before MCAO, during the occlusion, and during reperfusion (p<0.001 vs. Control Vehicle, **Fig. 4**). Furthermore, t-AUCB treated HFD+STZ/NA mice had decreased blood glucose levels compared to vehicle-treated HFD+STZ/NA mice at each time point (p<0.01 vs. HFD+STZ/NA Vehicle, **Fig. 4**).

**Table 1:** Baseline Characteristics of Type 2 Diabetic Mice and Controls.

	Control Vehicle	Control t-AUCB	HFD+STZ/NA Vehicle	HFD+STZ/NA t-AUCB
**Age (weeks)**	16±0.5	16±0.5	16±0.5	16±0.5
**Weight (g)**	26.0±0.8	25.6±0.8	26.8±0.9	25.7±0.8
**Temp (°C)**	36.5±0.5	36.5±0.5	36.5±0.5	36.5±0.5

Age, weight, and body temperature, monitored via rectal temperature probe, were measured in control and HFD+STZ/NA mice treated with vehicle or t-AUCB treatment (n = 11–16 mice per group).

### Cerebral Blood Flow during Reperfusion is Decreased in Type 2 Diabetic Mice and Rescued with sEH Inhibition

**Figure 5:**
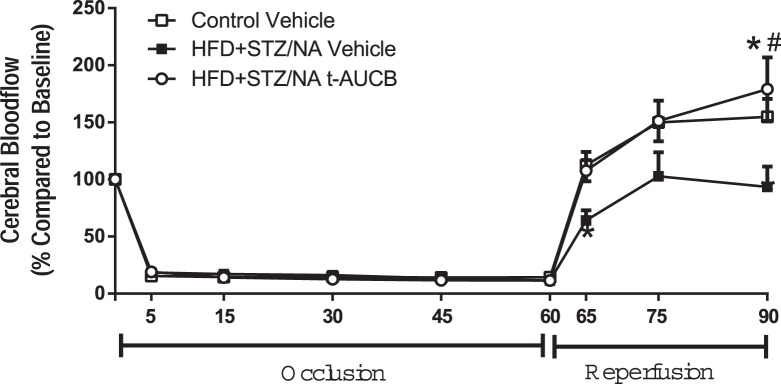
Cerebral Blood Flow during Reperfusion is Decreased in Diabetic Mice and Rescued with sEH Inhibition. Control and HFD+STZ/NA mice were treated with the sEH inhibitor t-AUCB (1 mg/kg i.p. daily, 7 days) or vehicle and subjected to middle cerebral artery occlusion (60 min). Relative perfusion, measured by laser Doppler probe, of the MCA territory measured continuously (averages of 5–15 min intervals are shown) at baseline, during the occlusion, and during 30 min post-reperfusion. *p<0.001 vs. Control Vehicle, #p<0.01 vs. HFD+STZ/NA Vehicle. N = 4–5 per group.

**Fig. 5** shows that relative perfusion, measured by laser Doppler probe, of the MCA territory after reperfusion was decreased in HFD+STZ/NA mice compared to controls (p<0.001 vs. Control Vehicle). Furthermore, sEH inhibition with t-AUCB prevented the perfusion deficit of the MCA territory during reperfusion in HFD+STZ/NA mice (p<0.01 vs. HFD+STZ/NA Vehicle).

### sEH Inhibition Prevented Type 2 Diabetes-induced Increases in Infarct Size following MCAO

**Figure 6:**
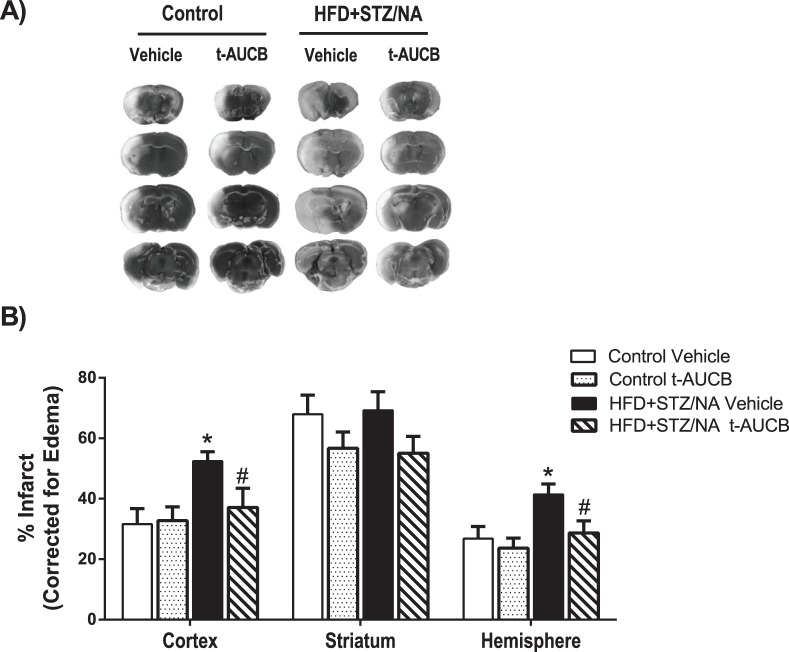
sEH inhibition prevented type 2 diabetic-induced increases in infarct size following MCAO. Control and HFD+STZ/NA mice were treated with the sEH inhibitor t-AUCB (1 mg/kg i.p. daily, 7 days) or vehicle and subjected to middle cerebral artery occlusion (60 min). Brains were harvested at 24 h post-MCAO, and infarct size measured by TTC staining and corrected for edema as described in methods. A) Representative images of infarct size in each treatment group. B) Infarct size is shown for separately for cortex, striatum, and total hemisphere. *p<0.05 vs. Control Vehicle, #p<0.05 vs. HFD+STZ/NA Vehicle, N = 8–10 per group.

**Figure 7:**
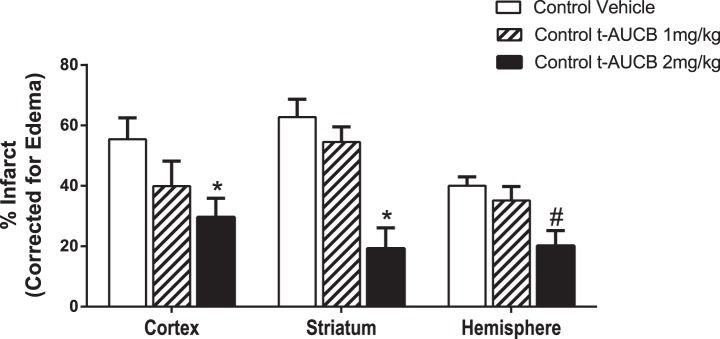
2 mg/kg t-AUCB prevented reduced infarct size in control mice following MCAO. Control mice were treated with the sEH inhibitor t-AUCB (1 mg/kg or 2 mg/kg i.p. daily, 7 days) or vehicle and subjected to middle cerebral artery occlusion (60 min). Brains were harvested at 24 h post-MCAO, and infarct size measured by TTC staining and corrected for edema as described in methods. Infarct size is shown for separately for cortex, striatum, and total hemisphere. *p<0.05 vs. Control Vehicle of same brain region via 2-way ANOVA post-hoc test, #p<0.01 vs. Control Vehicle Hemisphere via t-test only, N = 7 per group.

**Fig. 6** shows that HFD+STZ/NA vehicle-treated mice sustained a 66% larger cortical infarct size and 55% larger total hemisphere infarct size than control vehicle-treated mice (p<0.05). To test whether inhibition of sEH in HFD+STZ/NA-treated mice might decrease vulnerability to stroke, we blocked sEH activity using the specific antagonist t-AUCB during the week before MCAO. As stated above, treatment with t-AUCB decreased fasting glucose levels at baseline and throughout ischemia and improved reperfusion in the MCA territory in HFD+STZ/NA mice. In line with these improvements in glycemic status and cerebral perfusion, t-AUCB reduced cortical infarct size by 29% and total hemisphere infarct size by 31% in HFD+STZ/NA mice (p<0.05 vs. HFD+STZ/NA Vehicle, **Fig. 6**). Although the 1 mg/kg t-AUCB dose was not sufficient to reduce infarct size in control mice, a 2 mg/kg t-AUCB dose did decrease infarct size in control mice compared to vehicle treated control mice in the cortex, the striatum, and the total hemisphere (p<0.05 vs. HFD+STZ/NA Vehicle, **Fig. 7**).

## Discussion

The goal of this study was to determine if sEH inhibition, used as a preventative treatment, could provide protection against ischemic brain injury in type 2 diabetic mice. Previous studies have shown that several animal models of diabetes increase infarct size following cerebral ischemia [7]–[10], as we also observed in the present study. Using a long-term high fat diet rodent model of pre-diabetes, our data show that pre-diabetes increases expression of sEH mRNA and protein in brain, but does not significantly alter EETs levels in brain. Using a rodent model of type 2 diabetes that combines high fat diet with STZ/NA treatment, we further demonstrated that sEH inhibition with t-AUCB decreases blood glucose levels, increases cerebral perfusion, and decreases infarct size in type 2 diabetic mice. Our findings show that sEH inhibition, used as a preventative treatment, improves glycemic status, post-ischemic reperfusion in the ischemic territory, and stroke outcome in the setting of type 2 diabetes. However, based on the current study we cannot determine the relative contribution, if any, of improvements glycemic status and cerebral perfusion on stroke outcome. Future studies will address this issue by experimentally controlling for blood glucose levels between groups by co-administering t-AUCB with Metformin.

The current data support a novel role for sEH in both pre-diabetes and type 2 diabetes. We have previously shown that hyperglycemia, caused by beta cell destruction with STZ, increases EPHX2 expression in cerebral vessels and decreases EETs levels in brain [8]. However, it was unclear if this effect could be elicited by factors involved in the etiology of type 2 diabetes, other than hyperglycemia. We used a long-term high fat diet to model pre-diabetes, a diet which increases body weight and impairs glucose tolerance in the absence of overt resting hyperglycemia [19]. The HFD mice exhibited hyperinsulinemia, which likely preserved euglycemia in the absence of a glucose challenge. A similar phenomenon is observed clinically in patients with pre-diabetes. The first stage of pre-diabetes involves a long period of insulin resistance accompanied by a compensatory increase in pancreatic insulin secretion and increased beta cell mass [25]. During this phase, fasting glycemic status is normal, yet glucose intolerance can be unmasked in these patients with a glucose tolerance test (GTT). As the disease progresses, the increased insulin secretion progressively fails to compensate for the insulin resistance and hyperglycemia ensues over time. Eventually, the beta cells cannot produce enough insulin and hyperglycemia (type 2 diabetes) results [25]. Using the HFD model, we show that pre-diabetes causes increased EPHX2 gene expression as well as increased sEH protein expression in brain. These data indicate that sEH is elevated before the onset of type 2 diabetes. Surprisingly, despite increased mRNA and protein levels of sEH, we did not detect a statistically significant difference in EETs levels between chow and HFD fed mice. In fact, there was a trend for higher EETs in mice on HFD vs. normal chow. Because the level of EETs is regulated both by their synthesis via P450 epoxygenase and their metabolism via sEH, it is possible that EETs synthesis was increased to compensate for an increase in sEH expression. In a similar model, De Taeve et al. found that sEH mRNA and protein levels did not differ, but sEH activity was higher in adipose tissue from obese mice on “western-style” vs. regular diet [26]. If so, increased vulnerability to stroke injury in rodents fed a high fat diet, either in the presence (as we have observed) or absence of STZ/NA (as others have observed [27]), may not necessarily be the result of lower brain EETs, but rather to differences in insulin resistance and glycemic status. It is important to note that our model of pre-diabetes included impaired glucose tolerance. Clinical studies in pre-diabetic patients with impaired glucose tolerance show that these patients are at increased risk for stroke, while studies that do not include impaired glucose tolerance in their definition of pre-diabetes show less of an association [28].

Individuals with diabetes have more than twice the risk for stroke compared to non-diabetic individuals [1]. The mechanisms underlying increased ischemic sensitivity during the evolution of type 2 diabetes are poorly understood and no therapeutic options are currently available to reduce risk, other than weight reduction and glycemic control. Although hyperglycemia is associated with poor stroke outcome in both humans [2]–[4] and rodents [5]–[10], tight glycemic control in hyperglycemic patients has failed to protect against stroke incidence or improve outcome in clinical trials [11]–[16]. Therefore, the goal the current study was determine if sEH inhibition is a potential therapeutic target for type 2 diabetic stroke.

Our current data confirm that type 2 diabetes, induced by high fat diet combined with STZ/NA treatment, enhances ischemic injury in multiple brain regions. In addition we show that type 2 diabetes also causes a decrease in perfusion to the ischemic territory following MCAO, suggesting that vascular mechanisms are contributing to the enhanced susceptibility to ischemic injury in the context of type 2 diabetes. This is in contrast to our previous findings using a model of type 1 diabetes, induced by STZ, in which we found that CBF and cortical perfusion were unaltered in type 1 diabetic mice [8] compared to controls. In contrast to our previous type 1 diabetes model in which STZ is administered alone, in the current study nicotinamide, a B3 vitamin and precursor to NAD+, was used to reduce the toxicity of STZ and prevent large reductions in beta cell mass in mice fed a high fat diet to more closely model type 2 diabetes[20]. The HFD+STZ/NA mice did not exhibit a difference in circulating insulin concentration compared to controls, most likely due to pancreatic beta cell damage, as indicated by their relative hyperglycemia compared to controls. Nakamura et al. have shown that insulinemia is not altered by STZ/NA [20]. A similar phenomenon is observed clinically in many type 2 diabetic patients, in which the pancreatic beta cells progressively fail to secrete adequate insulin to maintain normal glycemic status, at which time circulating insulin concentration is not elevated [24], [25]. However, it should be noted that nicotinamide alone could also have had neuroprotective effects through multiple mechanisms. Nicotinamide has been shown to cross the blood brain barrier and to improve mitochondrial function/ATP synthesis following ischemia by acting as a PARP inhibitor [29]. Furthermore, nicotinamide has also been shown to protect against ischemic damage through the sirtuin-1 pathway [30]. However, these effects are unlikely to have contributed substantially to ischemic outcome in the current study, as the nicotinamide was administered two weeks prior to ischemia and the STZ/NA group had larger, not smaller, infarct size compared to controls. Nevertheless, important differences underlying etiology of type 1 and type 2 diabetic stroke may exist and warrant further investigation.

Other studies support our finding that vascular mechanisms may contribute to enhanced injury in type 2 diabetic mice. For example, using the db/db genetic model for type 2 diabetes, it was found that diabetic male mice have decreased cerebrovascular diameter, arteriolar density, and decreased blood brain barrier function in the ischemic brain compared to wild type mice [31]. Using the Goto Kakaizaki rat model for type 2 diabetes, it has also been shown that diabetes decreases vascular volume in the ischemic cortex and striatum [32]. Taken together, these data provide evidence that type 2 diabetic rodents have enhanced injury after stroke that is mediated, in part, by vascular dysfunction.

We have previously shown that sEH inhibition protects the brain from ischemic injury by a mechanism linked to preservation of EETs in non-diabetic mice [22], and more recently in type 1 diabetic mice [8]. Our current data show that sEH inhibition also reduces infarct size in type 2 diabetic mice. Interestingly, t-AUCB reduced infarct size in type 2 diabetic mice at a lower dose (1 mg/kg) than the dose that was needed in control mice (2 mg/kg) to elicit the same protective effect, suggesting that diabetic mice may be more sensitive to the protective effects of sEH inhibition. However, since we did not monitor serum levels of t-AUCB in either cohort, a difference in pharmacokinetics between groups cannot be ruled out. Another limitation of the current study is that we did not assess functional outcomes of sEH inhibition following stroke. In a previous study [22], we have shown that another sEH inhibitor, AUDA-BE, was effective in reducing infarct when administered after stroke. In the current study, sEH inhibitor was administered before stroke, which has limited value as a stroke therapy, but is ideal for determining the role of sEH in the exacerbation of stroke injury in brains of type 2 diabetic mice, which was the goal of the current study. Based on previous studies in non-diabetic mice, it is possible that sEH inhibitors could also attenuate neurological deficits following stroke [33], and we intend to address this possibility in future studies. Nevertheless, we show that sEH inhibition improves both glycemic status and cerebral perfusion in the ischemic territory in type 2 diabetic mice when administered as a pre-treatment. This is in contrast to our findings in type 1 diabetic mice, in which sEH inhibition protected against ischemic damage without altering glycemic status or early (5 min) re-perfusion to the ischemic territory [8]. Therefore, sEH inhibition appears to protect against type 1 and type 2 diabetic stroke, at least in part, through different mechanisms. EETs can provide neuroprotection by multiple mechanisms including: enhancing vasodilation [17], inhibiting of platelet adhesion [34], reducing inflammation [35], reducing of oxygen free radicals [36], reducing apoptosis, or activating protective signal transduction [17], [37]. In line with this, we have previously shown that sEH knockout mice are protected against ischemic injury in part due to enhanced cerebral blood flow after MCAO [38]. It has also been shown that EETs reduce ischemic injury by improving cerebrovascular structure and increasing microvascular density in spontaneously hypertensive rats [33].

In addition to the protective effects of EETs outlined above, EETs may have additional protective effects specifically in the context of diabetes. Although we did not observe differences in brain EETs levels in mice fed a high fat diet, sEH inhibition could still be more protective in type 2 diabetic mice than control mice because it could increase EETs in tissues other than the brain, especially the pancreas, which as previously reported by Luria et al. [39], would improve beta cell survival, insulin secretion and glycemic status. In line with this, we observed improvements in glycemic status consequent to sEH inhibition that could possibly have contributed to reductions in infarct size and cerebral perfusion. Since hyperglycemia is associated with poor stroke outcome in both humans [2]–[4] and rodents [5]–[10], any reduction in blood glucose levels may have also contributed to a reduction in infarct size. Furthermore, hyperglycemia has been shown to negatively affect cerebral vascular function and cerebral blood flow after ischemia [40], [41] in some studies, although others have shown no effect [42], [43]. Nevertheless, randomized prospective clinical trials have consistently failed to demonstrate that tight glycemic status improves outcome from stroke [12]–[16]. Similar to the reduction in blood glucose levels, in the HFD+STZ/NA mice, t-AUCB caused a 17% reduction in post-ischemic insulin levels compared to vehicle-treated HFD+STZ/NA, although this effect was not statistically significant. Likewise, enhancing EETs has been shown to enhance insulin signaling and improve insulin sensitivity [39], [44]–[46]. Luo et al. showed that either deletion of the sEH-coding gene or pharmacological inhibition of sEH attenuated hyperglycemia in STZ-treated mice, a model for type 1 diabetes [46]. They found that sEH inhibition improved STZ hyperglycemia by directly limiting STZ-mediated damage to pancreatic β-cells [46]. Luria et al. demonstrated that either sEH inhibition or sEH gene deletion reduces the severity of insulin resistance in mice fed a HFD [39]. Specifically, they found that sEH inhibition/gene deletion increased insulin receptor signaling, pancreatic islet size and vascularization, glucose clearance during a glucose tolerance test (GTT), and insulin sensitivity during an insulin tolerance test. Thus, both the availability of insulin and the peripheral tissue response to the insulin were both improved by limiting the influence of sEH. They further showed that sEH gene deletion or sEH inhibition yielded fasting glycemia that was lower during a high-fat diet when compared to control animals on the same diet. All of these effects occurred without a reduction in body mass or adiposity, indicating that these are primary effects [39]. This improvement in glucose homeostasis plays a role in the ability of EETs to protect against diabetic cardiomyopathy [45]. Furthermore, glycemic control can improve vascularization, which is needed for brain repair, in the ischemic cortex and striatum in type 2 diabetic mice and enhance sensorimotor and cognitive function recovery [32]. Future studies will determine if the ability of sEH inhibition to improve glycemic control translates to enhanced repair and recovery following cerebral ischemia via vascular mechanisms, such as enhanced vascularization in the ischemic territory.

In summary, we found that 1) pre-diabetes, induced by long-term high fat diet, increases expression of sEH mRNA and protein but not EETs levels in brain, 2) type 2 diabetes, induced by a combination of high fat diet and STZ/NA, exacerbates cerebral infarct size after MCAO in mice, and 3) sEH inhibition with t-AUCB, when administered prior to the ischemic event, improves glycemic status, increases cerebral perfusion, and reduces brain infarct size after MCAO. These findings indicate sEH inhibition improves stroke outcome, glycemic status, and post-ischemic perfusion in the ischemic territory in setting of type 2 diabetes. This data suggest that sEH inhibition may be a potential preventative therapeutic target for improving stroke outcome in individuals with type 2 diabetes who are at increased risk for stroke [1].

## Acknowledgments

The authors would like to thank the OHSU Bioanalytical Shared Resource/Pharmacokinetics Core for performing the EETs assay.
